# Mechanistic insights into viral−host interactions during transcription

**DOI:** 10.52601/bpr.2026.250066

**Published:** 2026-10-31

**Authors:** Zehong Huang, Tingyu Peng, Zirui Li, Guoliang Zhu, Zixiang Zhu

**Affiliations:** 1 State Key Laboratory of Animal Disease Control and Prevention, College of Veterinary Medicine, Lanzhou University, Lanzhou Veterinary Research Institute, Chinese Academy of Agricultural Sciences, Lanzhou 730000, China; 2 Gansu Province Research Center for Basic Disciplines of Pathogen Biology, Lanzhou 730046, China

**Keywords:** Transcriptional regulation, DNA virus, RNA virus, Virus-host interaction

## Abstract

Viral transcription strategies vary significantly depending on the nature of the viral genome and the site of transcription within the host cell. A common challenge for all viruses is to circumvent host defense mechanisms while simultaneously exploiting host cellular machinery to facilitate their own transcription. DNA viruses and retroviruses often hijack the host’s RNA polymerase II (Pol II), while RNA viruses typically encode their own RNA-dependent RNA polymerase (RdRp). Through these diverse mechanisms, viruses effectively manipulate host transcription, translation, and immune responses, thereby evading immune surveillance and completing their replication cycle. This article systematically reviews the transcription strategies employed by major DNA and RNA virus families, providing insights that contribute to a deeper understanding of viral transcriptional regulation mechanisms.

## INTRODUCTION

Transcription is a critical step in the viral life cycle. While positive-sense single-stranded RNA (ssRNA) viruses can undergo direct translation, they must still transcribe a negative-sense RNA strand to replicate their genomes (Köppke *et al.* 2024). DNA viruses that replicate in the nucleus (Braspenning *et al.* 2020; Burton *et al.* 1986; de Faria *et al.* 2022) and retroviruses (D’Orso 2025; Musier-Forsyth *et al.* 2024) possess genetic material structurally similar to the host genome, enabling them to exploit the host’s Pol II machinery. Pol II is a DNA-dependent RNA polymerase responsible for mRNA transcription in eukaryotes (Farnung 2025). Retroviruses represent a unique case among Pol II-utilizing viruses, as they reverse transcribe their RNA genome into DNA and integrate it into the host chromatin to commandeer Pol II. In contrast, cytoplasmic DNA viruses, such as poxviruses, encode their own viral RNA polymerase (vRNAP) (Broyles 2003). Since Pol II cannot use RNA as a template (Farnung 2023), most RNA viruses encode their own RdRp, which is essential for both genome replication and mRNA synthesis (Kaundal *et al.* 2024). Nevertheless, these viruses can still co-opt host RNA modification systems, such as capping and polyadenylation (de la Peña *et al.* 2007). This review briefly addresses the strategies and mechanisms viruses use to navigate the challenges of host factor regulation, including immune evasion and utilization of host transcriptional systems.

## VIRUSES LACKING VIRAL RNA POLYMERASE HIJACK HOST POL II

### Porcine parvovirus transcription

Many DNA viruses lack a vRNAP and instead enter the nucleus to utilize host Pol II for transcription. Virus families such as *Papillomaviridae*, *Polyomaviridae*, *Herpesviridae*, and *Parvoviridae* employ this strategy. This section uses porcine parvovirus (PPV) and pseudorabies virus (PRV) as examples to illustrate the transcriptional regulation mechanisms of DNA viruses lacking vRNAP.

A critical prerequisite for this host-dependent transcription is the nuclear import of the viral genome. Parvovirus capsids, with a diameter smaller than the nuclear pore complex (NPC) (Fay and Panté 2015), can enter the nucleus intact. The prevailing model involves two potential mechanisms. The first, well-supported mechanism involves pH-dependent conformational changes of the capsid within the endosome, which expose nuclear localization signals (NLSs) located within the unique N-terminal region of VP1 (Mani *et al.* 2006; Suikkanen *et al.* 2002). These NLSs, often resembling classical basic amino acid clusters, are recognized by importin α/β receptors, facilitating ATP-dependent transport through the NPC (Ihalainen *et al.* 2007; Lange *et al.* 2007) (Fig. 1A). A second, proposed mechanism suggests that interactions between the capsid and NPC-associated proteins could trigger extensive capsid rearrangements and potentially induce local disintegration of the nuclear envelope, facilitating capsid entry (Fig. 1B). Following nuclear import, the parvovirus genome is uncoated. While the precise mechanism remains elusive, evidence suggests that uncoating occurs at or post-entry without full capsid disassembly. Instead, subtle conformational changes allow the 3’ end of the viral single-strand DNA (ssDNA) to be extruded from an otherwise intact capsid, enabling it to serve as a primer for the host DNA polymerase to initiate replication and generate the transcriptional template. In summary, parvoviruses deliver their genomes into the nucleus to enable transcription by mediating nuclear entry through VP1 interactions or potentially by inducing localized rupture of the nuclear envelope.

**Figure 1:**
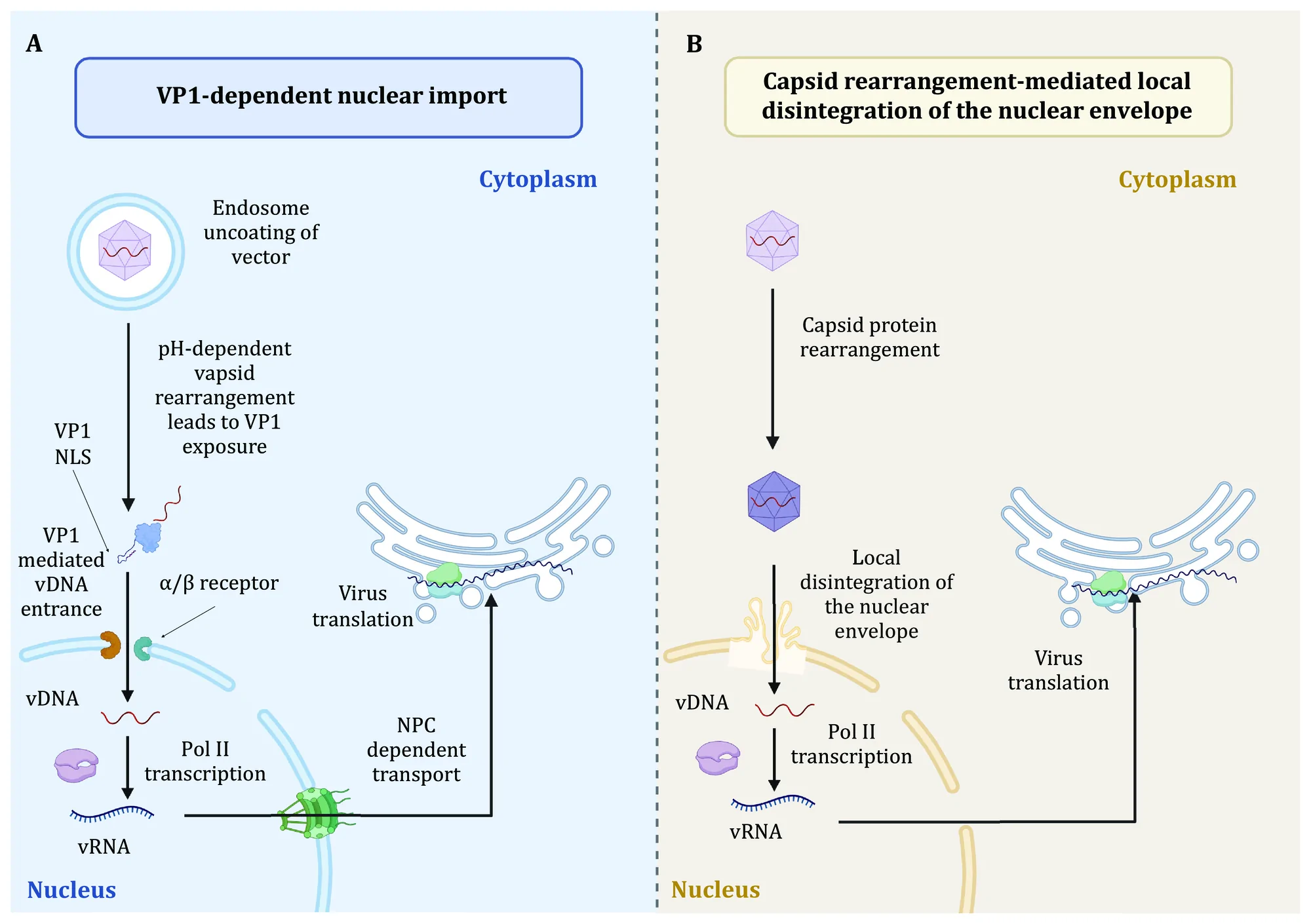
Models for nuclear import of the porcine parvovirus genome. **A** NPC-dependent import. The endosomal acidification triggers a conformational change in the VP1, exposing the nuclear localization signals (NLSs). The NLSs are recognized by the importin α/β heterodimer, facilitating the ATP-dependent transport of the intact capsid through the nuclear pore complex (NPC). The ssDNA genome is subsequently extruded for replication and transcription. **B** NPC-independent import. The viral capsid interacts with proteins at the nuclear envelope, potentially inducing local disruption or degradation of the nuclear membrane, thereby creating a passage for the capsid or viral genome to enter the nucleus. This figure was created using BioRender.com

### Pseudorabies virus transcription regulation

In contrast to parvovirus, herpesvirus transcription exhibits classic temporal regulation, as exemplified by PRV. Upon infection, the PRV genome enters the host nucleus and engages in extensive interactions with the host genome. The PRV genome shows a strong preference for associating with open chromatin regions and transcriptionally active zones enriched with Pol II, thereby creating a favorable environment for hijacking host Pol II. These virus−host interaction hotspots are characterized by a conserved binding motif for the host nuclear DNA-binding protein RUNX1, which serves as a critical bridge mediating chromatin interaction between PRV and the host. By positioning signals for Pol II recruitment near RUNX1 binding sites, the PRV genome effectively hijacks host Pol II to facilitate viral transcription. This mechanism highlights the RUNX1 binding site as a promising target for anti-PRV strategies (Xiao *et al.* 2021).

After the fusion of the PRV envelope with the cell membrane, the capsid and tegument proteins are released into the cytoplasm. Viral tegument proteins immediately begin to modulate the host cell’s protein synthesis machinery and initiate a temporally ordered viral transcription cascade directed by RNA Pol II, a molecular hallmark of herpesvirus infection. PRV genes are subdivided into at least three distinct classes of consecutively expressed transcripts: immediate-early (IE), early (E), and late (L) (Roizman and Knipe 2001) (Fig. 2). Viral transcriptional activators, expressed within the first one to 2-h post-infection, drive the transcription cascade by activating the next set of viral genes. The promoter of the herpesvirus immediate-early gene is recognized by host transcription factors and Pol II, leading to the synthesis of IE proteins such as IE180 directly after infection. Meanwhile, in addition to Pol II, herpesvirus infection also hijacks topoisomerase I (TOP1), which consequently induces host chromatin condensation, suppresses host gene expression, and diverts transcriptional resources (González-Almela *et al.* 2025). The expression of early genes, which encode proteins necessary for DNA replication and nucleotide metabolism, requires viral transcriptional-associated factors encoded by immediate-early genes. Efficient transcription of true late genes, which often encode structural proteins for virion assembly and egress, requires viral DNA replication. The mechanistic details of the transition from early to late gene transcription in PRV-infected cells remain incompletely understood (Pomeranz *et al.* 2005). In brief, herpesvirus replication is characterized by a temporally regulated transcriptional cascade, initiating with immediate-early and early gene expression, followed by viral DNA replication, which triggers late gene transcription.

**Figure 2:**
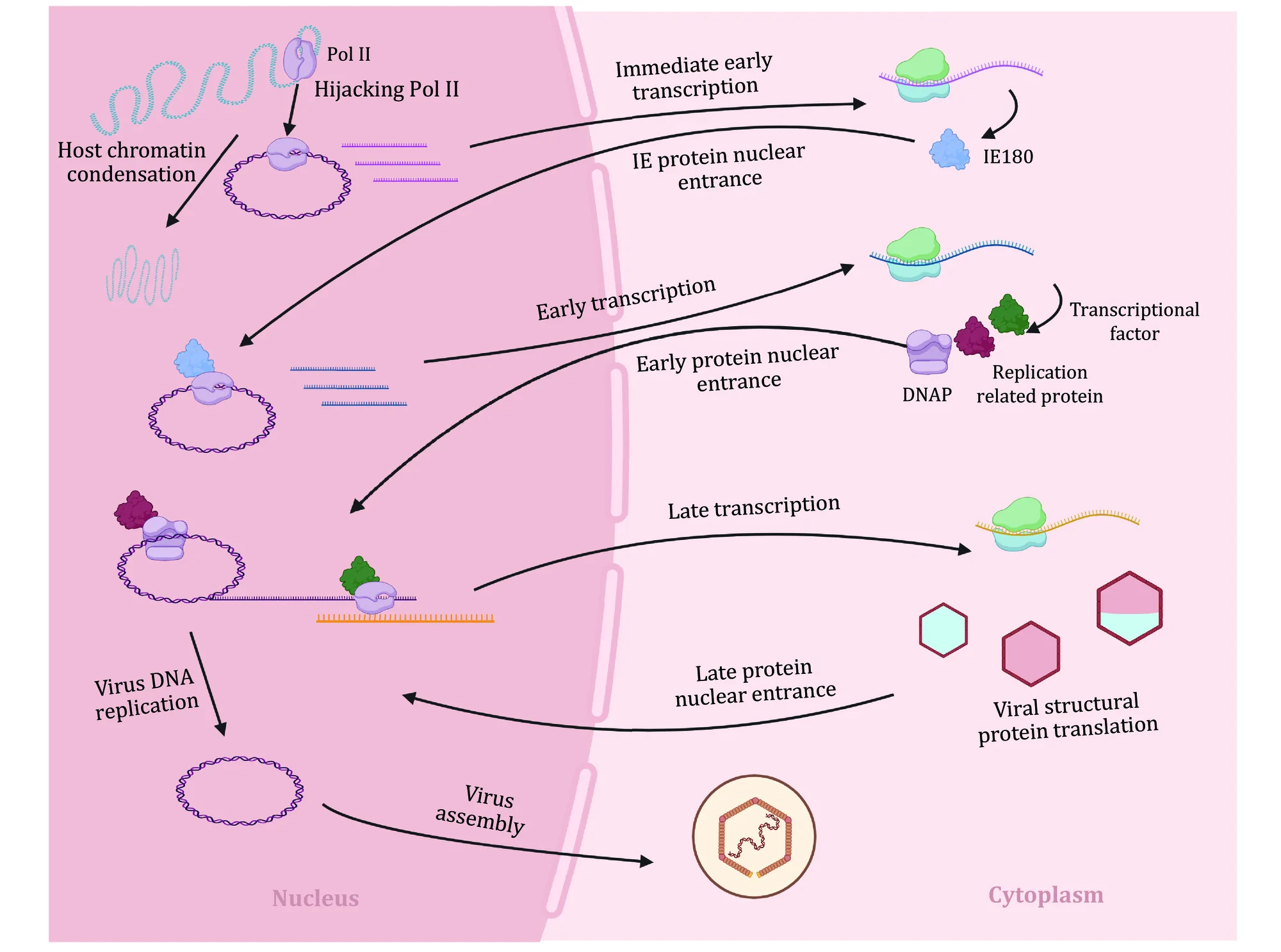
Temporal regulation of herpesvirus (pseudorabies virus) transcription. Herpesvirus gene expression follows a sequential cascade, divided into immediate-early (IE), early (E), and late (L) phases. The transcription of each gene class depends on proteins synthesized in the preceding phase. IE genes, encoding regulatory proteins, are transcribed immediately upon infection by host RNA polymerase II (Pol II). Concurrently, the virus hijacks host topoisomerase I (TOP1), inducing host chromatin condensation and diverting transcriptional resources. E genes, including viral DNA replication enzymes, require IE protein trans-activators for their expression. The onset of viral DNA replication (dashed vertical line) triggers the robust transcription of L genes, which primarily encode structural components for virion assembly and egress. This figure was created using BioRender.com

### Hepatitis D virus transcription

Unlike most cytoplasmic RNA viruses, hepatitis D virus (HDV) uniquely exploits host Pol II for its nuclear replication. HDV possesses a single-stranded RNA genome of approximately 1.7 kb that exhibits high self-complementarity, forming a rod-like structure that mimics double-stranded DNA. This structural mimicry allows the viral RNA to be recognized and transcribed by Pol II, enabling both mRNA synthesis and full-genome replication — an elegant adaptation reflecting HDV’s evolutionary ingenuity (Abbas and Afzal 2013; Urban *et al.* 2021). HDV infection and replication depend on the hepatitis B surface antigen (HBsAg) for virion assembly and release (Lempp *et al.* 2016; Lucifora and Delphin 2020).

The HDV genome encodes two isoforms of the hepatitis delta antigen (HDAg): the small form (S-HDAg) and the large form (L-HDAg). S-HDAg is essential for initiating and sustaining viral replication, whereas L-HDAg acts as a negative regulator of RNA synthesis and is required for virion packaging through interaction with HBsAg (Urban *et al.* 2021). Following nuclear import of the HDV ribonucleoprotein (RNP) complex, Pol II transcribes the viral mRNA, which is translated into S-HDAg. A specific post-translational modification — the addition of a tryptophan residue and a 19–20 amino acid C-terminal extension — converts S-HDAg into L-HDAg (Lucifora and Delphin 2020). Both antigens bind HDV RNA to form RNPs that support subsequent rounds of transcription and genome replication (Fig. 3).

**Figure 3:**
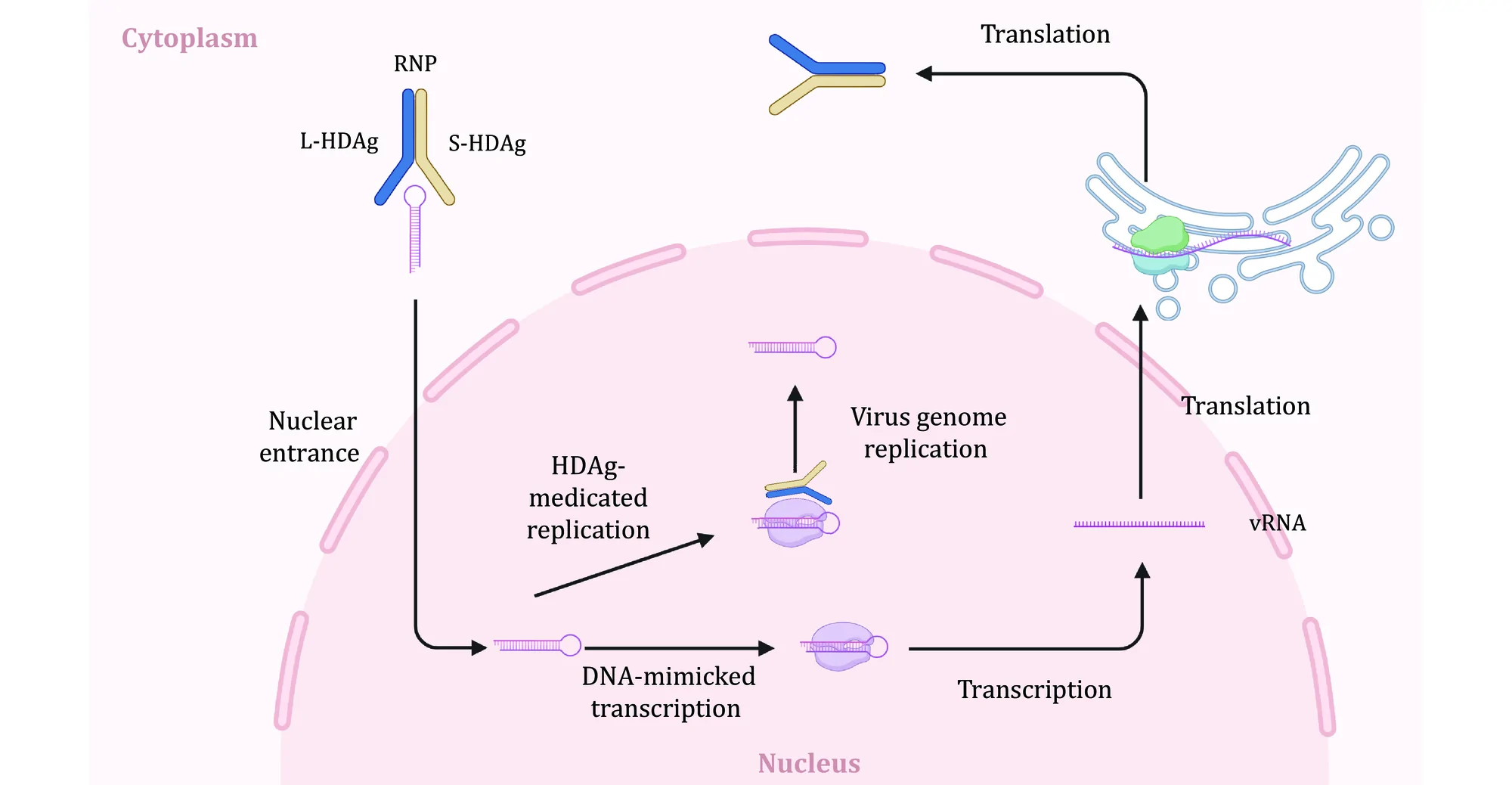
HDV transcription. Upon nuclear import of the ribonucleoprotein (RNP) complex composed of HDV RNA and both small (S) and large (L) forms of HDAg, the viral genome is transcribed by host RNA polymerase II (Pol II). The high self-complementarity of the HDV RNA confers a DNA-like structure, enabling Pol II to recognize it as a template. The viral mRNA is translated into S-HDAg, which is subsequently modified by the addition of a tryptophan and a C-terminal extension to yield L-HDAg. Both HDAg isoforms bind newly synthesized genomic RNA to form progeny RNPs, perpetuating the replication cycle. This figure was created using BioRender.com

### Retrovirus transcription

In contrast to most other RNA viruses, members of the *Retroviridae* family do not encode a vRNAP. Instead, they rely on reverse transcribing their RNA genome into DNA, integrating it into the host cell genome, and recruiting host Pol II through upstream *cis*-regulatory elements to drive their transcription. This section uses human immunodeficiency virus type 1 (HIV-1) (Fig. 4) and porcine endogenous retrovirus (PERV) as examples to illustrate retroviral transcriptional regulation strategies.

**Figure 4:**
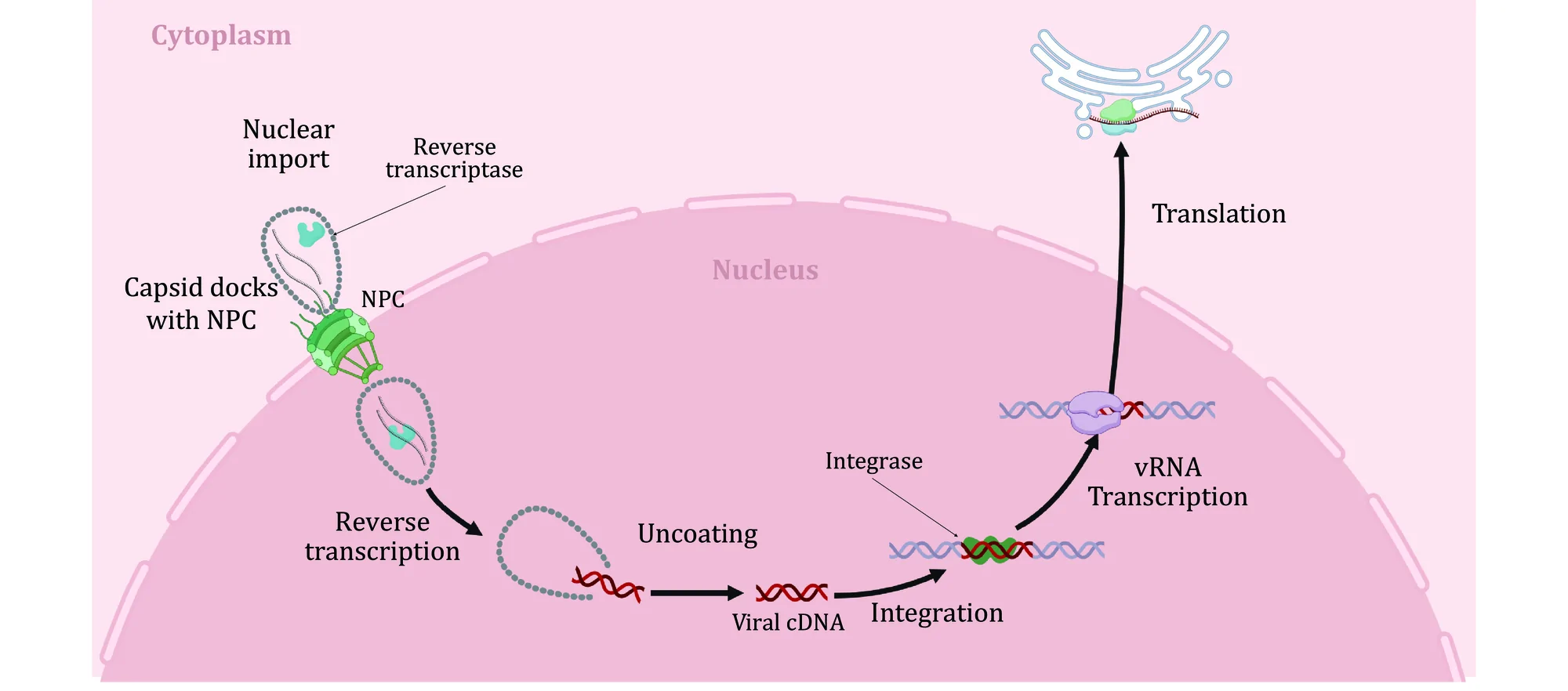
HIV-1 transcriptional activation from the integrated provirus. After docking at the nuclear pore complex (NPC) via the intact nucleocapsid, the HIV-1 core enters the nucleus, where reverse transcription is completed. The viral complementary DNA (cDNA) is then integrated into the host genome. Transcription is initiated from the 5’ long terminal repeat (LTR) promoter, where host transcription factors (*e*.*g*., NF-κB, Sp1) and the TATA-binding protein (TBP) recruit RNA polymerase II (Pol II) to transcribe viral RNA. The newly synthesized viral transcripts are exported from the nucleus to the cytoplasm for translation and subsequent virion assembly. This figure was created using BioRender.com

After reverse transcription and integration, HIV-1 gene expression is primarily accomplished by hijacking host Pol II. Transcription is initiated from the *cis*-regulatory promoter within the 5’ long terminal repeat (5’ LTR). This process is regulated by cellular transcription factors, and the establishment of a chromatin state conducive to HIV transcription involves modulation through epigenetic modifications (Colin and Van Lint 2009; Colin *et al.* 2014). The HIV-1 5’LTR is divided into the modulatory region, the enhancer, the core promoter, and the leader region (Gaynor 1992; Pereira *et al.* 2000). Numerous *cis*-regulatory elements within these regions bind transcription factors to regulate HIV transcription. Among these, NF-κB, Sp1, and the TATA-box binding protein (TBP) are considered core elements for HIV transcription (Perkins *et al.* 1993; Suñé and García-Blanco 1995).

The enhancer region contains multiple NF-κB binding sites, underscoring its importance for initiating HIV transcription (Bachu *et al.* 2012). Under latent conditions, the NF-κB heterodimer p50/p65 is sequestered in the cytoplasm by interaction with IκBα. Upon activation of signaling pathways (*e*.*g*., PKC/MAPK), IκBα is phosphorylated by IκB kinase (IKK) and degraded, allowing p50/p65 to translocate into the nucleus. There, they bind the HIV-1 5’LTR and recruit histone acetyltransferases (HATs) to promote histone acetylation, facilitating HIV-1 transcription (Wang *et al.* 2018).

Sp1 binds to three GC-rich sites in the core promoter, downstream of the NF-κB sites (Gaynor 1992). Upon cellular stimulation, Sp1 acts synergistically with NF-κB to activate viral transcription (Perkins *et al.* 1993). This synergy involves interactions between NF-κB, Sp1, and Pol II transcription factors, including components of the general transcription factor TFIID (Guermah *et al.* 1998).

To recruit Pol II, the 5’LTR core promoter contains a non-canonical TATA box (CATA box, sequence CATATAA). This key DNA sequence is crucial for maintaining Pol II-dependent promoter activity. The TATA-binding protein (TBP), a subunit of TFIID, binds this site and induces the formation of the transcription pre-initiation complex. The sequence of this CATA motif exhibits diversity among different HIV-1 subtypes, which influences viral fitness (Dutilleul *et al.* 2020; van Opijnen *et al.* 2004).

Similar to HIV, PERV regulates transcription through transcription factors binding its LTR. The primary distinction lies in the LTR structure and the specific transcription factors recruited. PERVs are integrated into the swine genome (Denner and Tönjes 2012). As pigs are considered potential sources of donor organs for xenotransplantation, the risk of PERV transmission to humans is a concern (Denner *et al.* 2003). Human cells are susceptible to various PERV types (*e*.*g*., PERV-A, PERV-B, PERV-A/C) due to the presence of specific human receptors (huPAR1, huPAR2) (Denner and Tönjes 2012; Ericsson *et al.* 2003; Halecker *et al.* 2022; Patience *et al.* 1997; Specke *et al.* 2001). Although studies have investigated PERV transcriptional activation in human cells, the pathogenicity of activated PERVs remains unclear (Hossain *et al.* 2024).

## VIRUSES ENCODING VIRAL RNA POLYMERASE

### RNA-dependent RNA polymerase (RdRp)

The RNA-dependent RNA polymerase (RdRp) serves as the central enzyme for genome replication and mRNA transcription in most RNA viruses. Despite variations in full-length sequences and accessory domains that confer additional functionalities (*e*.*g*., methyltransferase, helicase), the catalytic core of RdRps is structurally conserved across many RNA virus families (Mönttinen *et al.* 2021). This core typically adopts a characteristic right-hand architecture, comprising palm, fingers, and thumb subdomains. The palm subdomain harbors conserved catalytic motifs (A–G) (Gao *et al.* 2020) responsible for nucleotide addition and metal ion coordination, while the fingers and thumb subdomains are crucial for template binding, NTP entry, and the stabilization of the RNA duplex (Gao *et al.* 2020).

SARS-CoV-2, the causative agent of COVID-19, provides a pertinent model for structural studies of viral RdRps (Fig. 5A). Its RdRp core is encoded by non-structural protein 12 (nsp12). Structural analyses reveal that nsp12 adopts the canonical RdRp fold. The active site within its palm domain is formed by conserved polymerase motifs A–G. Specifically, motif A (residues 611–626) contains the divalent cation-binding residue D618, which is critical for catalysis. The NTP entry channel is lined by hydrophilic residues, including K545, R553, and R555, within motif F. The RNA template strand enters the active site — composed of motifs A and C — through a groove clamped by motifs F and G. The primer strand is supported by motif E and the thumb subdomain, while the product-template hybrid exits via a dedicated RNA exit tunnel on the polymerase’s front face (Hillen *et al.* 2020; Wang *et al.* 2020).

**Figure 5:**
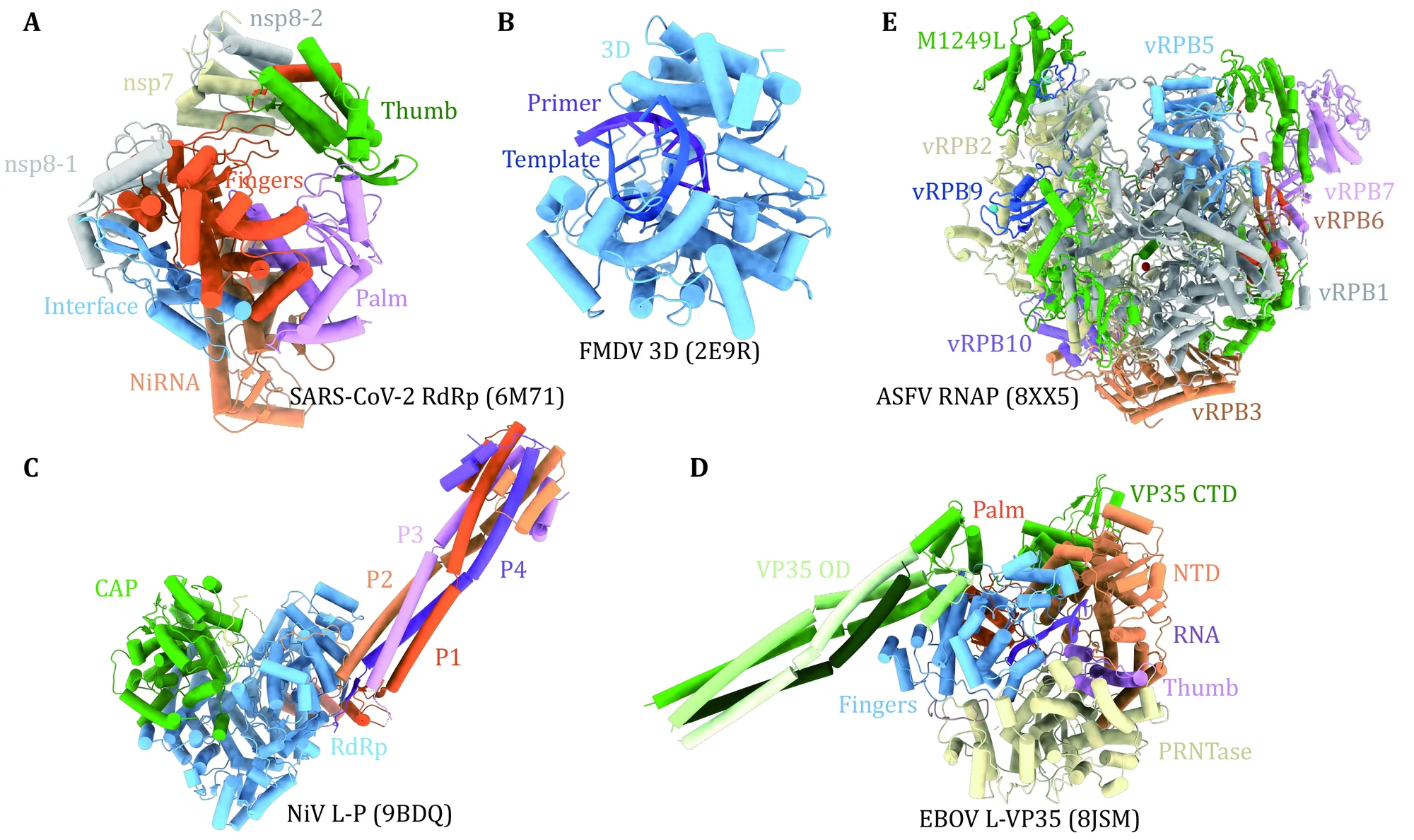
Representative structures of viral RNA polymerases. **A** SARS-CoV-2 RdRp (PDB: 6M71 (Gao *et al.* 2020) ). **B** FMDV 3D Polymerase (PDB: 2E9R (Ferrer-Orta *et al.* 2007) ). **C** Nipah virus (NiV) L−P complex (PDB: 9BDQ (Hu *et al.* 2025a)). **D** Ebola virus (EBOV) L-VP35 complex (PDB: 8JSM (Peng *et al.* 2023)). **E** African swine fever virus (ASFV) vRNAP (PDB: 8XX5 (Zhu *et al.* 2025))

A critical feature of the SARS-CoV-2 RdRp is its formation of a highly processive replication-transcription complex (RTC) by associating with essential cofactors nsp7 and nsp8. Nsp7 and two copies of nsp8 form a stable heterotetrameric complex (nsp7–(nsp8)_2_) that binds to the interface of the nsp12 thumb and finger domains, creating a unique circular assembly. This nsp7–nsp8 complex functions as a processivity clamp, dramatically enhancing the ability of nsp12 to synthesize long RNA products. Structural studies show that one nsp8 subunit, which possesses a C-terminal helix that extends towards the nsp12 NTP entry channel, may play a role in template positioning or NTP recruitment. The overall architecture of the nsp12–nsp7–nsp8 complex is essential for robust RNA synthesis and represents a prime target for antiviral therapeutics like remdesivir, which incorporates into the growing RNA chain within the active site (Kokic *et al.* 2021; Yin *et al.* 2020).

Another significant example is the RdRp of the foot-and-mouth disease virus (FMDV) (Ferrer-Orta *et al.* 2007), a member of the Picornaviridae family. The FMDV RdRp, known as the 3D protein (Fig. 5B), is located at the C-terminus of the viral polyprotein and only exhibits polymerase activity after proteolytic maturation from its precursors (Campagnola *et al.* 2008). Beyond its primary polymerase function, the FMDV 3D protein exhibits intricate interactions with host factors. Upon FMDV infection, the host nuclear RNA-binding protein Sam68 relocalizes from the nucleus to the cytoplasm. Sam68 acts as a mediator, assisting 3D in recruiting the viral 3C protein to enhance viral transcription efficiency (Lawrence *et al.* 2012; Rai *et al.* 2015a, b). The 3D protein coordinates extensive protein–protein and protein–RNA interaction networks, partly mediated by its nuclear localization signals (NLSs) (Kloc *et al.* 2020) and interactions with transcripts (Herod *et al.* 2016). Furthermore, FMDV 3D has evolved interferon (IFN)-suppressive capabilities. It can inhibit host antiviral responses by interacting with key molecules in the IFN pathway, such as IKKα, IKKε, IRF3, IRF7, NEMO, MDA5, and MAVS, thereby creating a favorable environment for viral replication (Sarry *et al.* 2023).

The structure of RdRp is highly conserved across viral genomes, which is crucial for its proper allosteric transitions and catalytic efficiency. This conserved architecture facilitates processive RNA synthesis, template binding, and NTP selection, while also serving as a target for antiviral drug design (Černý *et al.* 2014; Ramaswamy *et al.* 2022; Venkataraman *et al.* 2018; Wu *et al.* 2020). Through synonymous codon mutations in RdRp, SARS-CoV-2 can adapt to different hosts. For instance, the NSP12 P323L/G671S mutations enable SARS-CoV-2 variants to enhance viral replication in the upper respiratory tract of ferrets and increase transmissibility (Kim *et al.* 2023). Simultaneously, SARS-CoV-2 can employ a mechanism of nonsynonymous mutations to develop increased resistance to antiviral medicines (Stevens *et al.* 2022).

In summary, the RdRp structure is highly conserved among RNA viruses, comprising fingers, palm, and thumb subdomains. The palm subdomain harbors the catalytic activity, while the fingers subdomain is involved in template binding and factor recruitment. The distinct strategies employed by viruses like SARS-CoV-2 and FMDV — ranging from the precise architectural organization of the catalytic core and its essential cofactors to the sophisticated recruitment and neutralization of host factors — highlight the central role of RdRp in the viral life cycle and virus−host interactions.

### The Nipah virus (NiV) encodes a ladle-shaped polymerase complex

Negative-sense RNA viruses from the *Paramyxoviridae* and *Filoviridae* families utilize large polymerase proteins (L) that complex with cofactor proteins to form functional vRNAP assemblies. These complexes consistently exhibit a characteristic ladle-shaped architecture, demonstrating evolutionary convergence in transcriptional machinery organization.

The Nipah virus (NiV) L protein represents a multi-functional enzyme containing six structural domains: N-terminal domain (NTD) (Hu *et al.* 2025b), RNA-dependent RNA polymerase (RdRp) domain, phosphoprotein-binding domain (PRNTase), connector domain (CD), methyltransferase (MTase) domain, and C-terminal domain (CTD). Within the functional L−P complex, the L protein constitutes the catalytic “bowl” while the P protein forms the structural “handle.” The NTD establishes extensive interfaces with both PRNTase and RdRp domains, contributing to template/NTP channel formation and L−P complex stabilization. The RdRp domain maintains conserved fingers−palm−thumb topology, with finger elements directing complex assembly (Fig. 5C), palm regions housing catalytic centers and supporting structures, and thumb segments coordinating elongation processes. The PRNTase domain enables 5’ cap formation and guides RNA exit pathways (Wang *et al.* 2024; Yang *et al.* 2024).

The NiV P protein functions as a modular scaffold comprising four structural regions: a dynamic N-terminal domain (NTD), oligomerization domain (OD), flexible linker, and C-terminal X-domain (XD). The OD and XD mediate critical interactions with the L protein, while the XD additionally provides a binding interface for the viral nucleoprotein, thereby connecting transcriptional activity with ribonucleoprotein packaging (Hu *et al.* 2025b; Sala *et al.* 2025).

Ebola virus (EBOV) employs a structurally conserved approach through its L-VP35 complex (Fig. 5D). The characteristic ladle-shaped organization is maintained, with discernible L protein regions (NTD, RdRp, and PRNTase) adopting configurations parallel to paramyxovirus polymerases (Qi and Yi 2023). The RdRp domain contains six conserved catalytic motifs (A−F) arranged in canonical right-hand architecture. Functional transcription requires complex formation with tetrameric VP35, which contacts multiple L protein regions (fingers, palm, and NTD), emphasizing the cofactor’s indispensable role in complex stabilization and partner recruitment (Peng *et al.* 2023; Yuan *et al.* 2022).

During transcription by the ladle-shaped RNAP, the handle-like P protein or VP35 serves multiple functional roles: it links the L protein to the RNA template, recruits the nucleoprotein (N) to encapsidate newly synthesized RNA, thereby protecting it from host degradation. Meanwhile, the bowl-shaped L protein executes RNA synthesis, capping, and methylation. This structural arrangement enhances processivity by coordinating multiple enzymatic activities in a spatially organized manner, facilitates cofactor recruitment through modular interactions, and aids in immune evasion by shielding viral RNA from host sensors (Bloyet 2021; Horikami *et al.* 1992; Kleiner and Fearns 2024; Luo *et al.* 2020).

Collectively, these ladle-shaped polymerase complexes exemplify an evolutionarily refined structural paradigm for negative-sense RNA virus transcription. The catalytic “bowl” integrates RNA synthesis and processing activities, while the cofactor “handle” ensures structural coherence and connects the polymerase to viral replication components.

### DNA-dependent-RNA polymerase (DDRP)

Unlike DNA viruses that depend on nuclear host transcription machinery, nucleocytoplasmic large DNA viruses (NCLDVs) possess self-encoded multi-subunit DNA-dependent RNA polymerases (DDRPs). African swine fever virus (ASFV), a pathogen with substantial economic impact on global swine production, serves as a representative model of this autonomous transcriptional approach (Kuznar *et al.* 1980).

NCLDVs are characterized by their capacity to encode a complete transcription apparatus analogous to eukaryotic systems, featuring core polymerase subunits and associated transcription factors. Research on ASFV has identified numerous viral proteins involved in mRNA synthesis and processing (Cackett *et al.* 2020; Zhang *et al.* 2023). Initial structural characterization through recombinant expression identified an eight-subunit core complex (vRPB1, vRPB2, vRPB3-11, vRPB5, vRPB6, vRPB7, vRPB9, and vRPB10) as the fundamental functional unit (Pilotto *et al.* 2024; Zhu *et al.* 2025). Subsequent structural analyses of the native ASFV vRNAP revealed a more sophisticated assembly landscape, with five distinct compositional states categorized according to M1249L subunit occupancy: the core complex (CC) and M1249L C-tail occupied complexes 1−4 (MCOC1−MCOC4). M1249L engages seven core subunits to establish a stabilizing cage-like framework, with its C-terminal segment proving essential for both transcriptional competence and viral gene regulation (Fig. 5E). Structural conservation is evident among the core subunits: vRPB1 and vRPB2 form the catalytic center, while vRPB3-11 and vRPB10 create a rear platform structurally homologous to eukaryotic Pol II. The peripheral claw-like element consists of vRPB5, vRPB6, and vRPB7. Significantly, extended surface regions of vRPB2, vRPB3-11, vRPB10, vRPB7, and M1249L display virus-specific conformations. These distinctive structural attributes likely establish specialized interfaces for viral transcription factors, representing unique adaptations for host transcriptional manipulation (Zhu *et al.* 2025). This conserved subunit composition enables ASFV RNAP to adopt a eukaryotic-like architecture that supports efficient promoter recognition, initiation, and elongation. The virus-specific transcription initiation factors, which lack eukaryotic homologs, allow ASFV to directly recognize diverse viral promoters and potentially evade host transcriptional regulation mechanisms.

ASFV RNAP shares many similarities with Pol II (Iyer *et al.* 2001; Mirzakhanyan and Gershon 2017; Rodríguez and Salas 2013; Stevens *et al.* 2022): Pol II consists of 12 subunits, while ASFV RNAP encodes nine homologs of Pol II subunits, as well as homologs of the general transcription initiation factors (TBP /TFIIB) (Alejo *et al.* 2018; Rodríguez and Salas 2013; Salas *et al.* 1981; Yáñez *et al.* 1993), and the transcript cleavage/elongation factor (TFIIS). Additionally, ASFV encodes virus-specific transcription initiation factors with no eukaryotic homologs, which directly transcribe diverse viral promoters (Stevens *et al.* 2022; Yutin and Koonin 2012). In summary, transcription in ASFV more closely resembles that of Pol II.

## VIRAL−HOST TRANSCRIPTIONAL INTERACTIONS TARGETING HOST FACTORS

To ensure their own gene transcription and complete their life cycle, viruses employ various strategies to regulate both host and viral transcriptional processes. These include modulating the activity of host immune-related factors to evade innate immunity, mediating the stability of viral RNA (vRNA) or promoting the degradation of host mRNA, and facilitating membrane fusion to form replication compartments that shield viral processes from host factors (Fig. 6).

**Figure 6:**
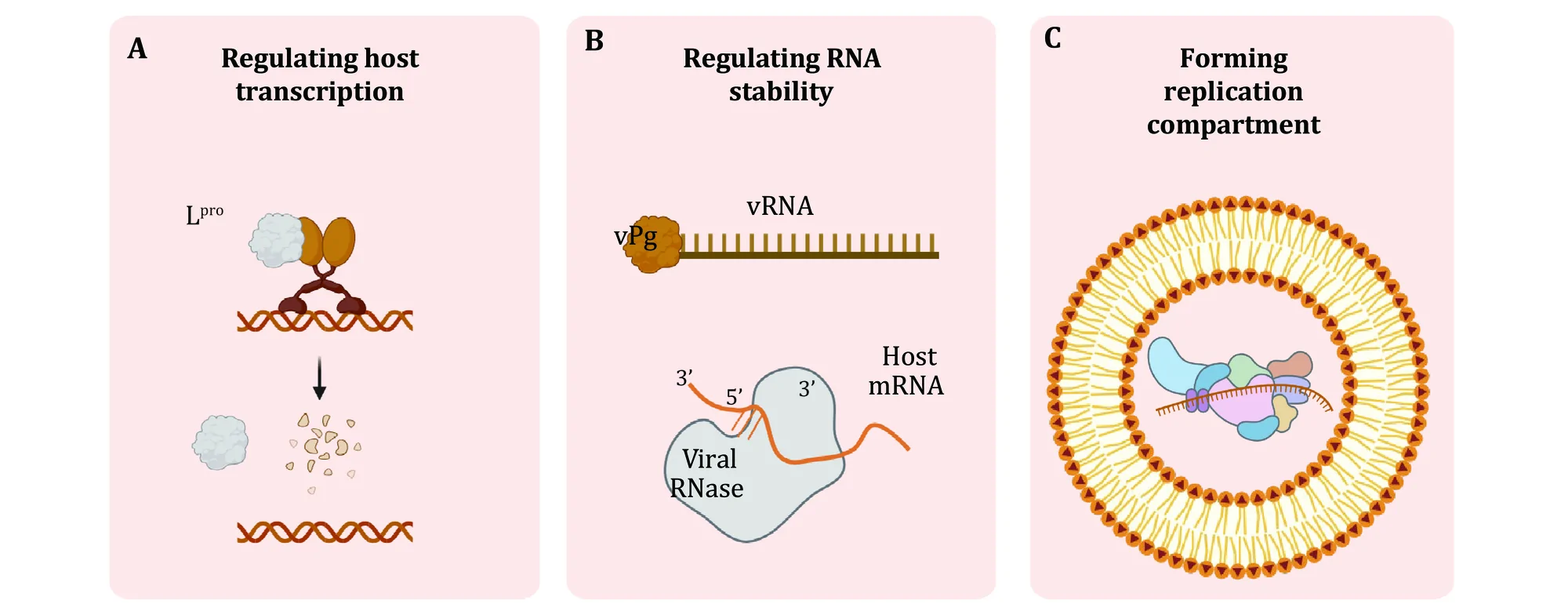
Strategies of viral-host interaction in transcription. Viruses employ multiple mechanisms to subvert host cellular processes and facilitate their own transcription. **A** Modulation of host transcription factors. Viral proteins can inhibit innate immune responses by degrading or inactivating key host transcription factors, such as the FMDV Lpro-mediated degradation of NF-κB p65. **B** Regulation of RNA stability. Viruses enhance the stability of their viral RNA (vRNA) through RNA modifications and by recruiting protective host factors (*e*.*g*., PEDV recruitment of Musashi homolog 1), while simultaneously promoting the degradation of host mRNAs (*e*.*g*., SARS-CoV-2 nsp1-induced endonucleolytic cleavage). **C** Formation of replication compartments. Viruses induce the remodeling of host membranes to create specialized compartments that shield viral replication and transcription from host defense sensors. Depicted is a SARS-CoV-2-induced double-membrane vesicle (DMV) housing the replication-transcription complex (RTC). This figure was created using BioRender.com

### Viral proteins modulate the activity of host transcription factors

The FMDV 3C protease, known for processing the viral polyprotein, also functions in regulating host transcription (Fig. 6A). In FMDV-infected cells, nuclear histone H3 is cleaved, yielding a novel chromatin-associated fragment termed Pi (Grigera and Tisminetzky 1984). This conversion is catalyzed by FMDV 3C protease. Since the cleaved fragment of H3 corresponds to a putative regulatory domain involved in modulating transcriptionally active chromatin, this specific cleavage may be associated with the host transcriptional shutoff observed in picornavirus infections, whereby 3C^pro^ indirectly regulates host transcription (Falk *et al.* 1990). This activity is influenced by the peptide sequence within the upstream 3A domain (Capozzo *et al.* 2002).

As a primary virulence factor, the FMDV leader protease (L^pro^) is the first protein translated. It regulates host immune-related gene transcription by degrading host transcription factors and exhibiting deubiquitinase activity. Following infection, activation of transcription factors like NF-κB, IRF3/7, and AP-1 induces IFN-α/β mRNA, establishing an antiviral state (Honda *et al.* 2005). Viruses have evolved mechanisms to suppress IFN induction and signaling (Conzelmann 2005; de los Santos *et al.* 2007; Haller *et al.* 2006).

L^pro^ degrades the NF-κB subunit p65/RelA, impairing the expression of IFN-β and inflammatory cytokines (de los Santos *et al.* 2007). It also modulates other transcription factors, including ISGF3G, IRF1, and IRF2, to regulate host innate immunity (Zhu *et al.* 2010). Furthermore, L^pro^ reduces IFN-α1/β expression by diminishing IRF-3/7 protein levels and downregulates transcription of multiple IRF-responsive genes (*e*.*g*., 2’,5’-OAS, ISG54). Thus, FMDV employs multiple strategies, via L^pro^, to counteract immune responses (Medina *et al.* 2017).

Additionally, L^pro^ ensures viral translation and transcription by degrading transcriptional and translational repressors. Gemin5, an RNA-binding factor of the survival of motor neurons (SMN) complex (Battle *et al.* 2006), binds the FMDV internal ribosome entry site (IRES) and downregulates viral translation (Pacheco *et al.* 2009). Furthermore, L^pro^ hydrolyzes Gemin5 to ensure viral transcription (Piñeiro *et al.* 2012), demonstrating its functional diversity and providing new insights into the survival competition between viruses and hosts. L^pro^ also recruits host factors like activity-dependent neuroprotective protein (ADNP), which suppresses IFN and ISG transcription, to promote viral replication (Medina *et al.* 2017). Beyond degrading transcription factors, L^pro^ possesses deubiquitinase activity that inhibits ubiquitination of key signaling molecules like RIG-I, TBK1, TRAF3, and TRAF6, thereby blocking type I IFN response activation (Wang *et al.* 2011).

In summary, FMDV employs a coordinated transcriptional strategy through its non-structural proteins: Lpro degrades or deubiquitinates key host transcription factors (*e*.*g*., NF-κB p65, IRF3/7) to suppress interferon responses, while 3C cleaves histone H3 to globally dampen host transcription. These activities work in concert to redirect cellular resources toward viral transcription and replication, exemplifying a multi-pronged approach to transcriptional hijacking and immune evasion.

### Mediating the stability of viral RNA and promoting the degradation of host mRNA

In the competition for cellular resources, viruses often stabilize their RNA and degrade host mRNA (Fig. 6B).

The FMDV 3B protein (VPg) acts as a protein primer for RNA synthesis. Unlike most picornaviruses with a single VPg copy, FMDV encodes three distinct copies (3B1, 3B2, 3B3), providing a replication advantage. Trans-replication of the FMDV replicon requires at least two 3B copies, reflecting the evolution of both cis and trans functions, also seen with the FMDV 3D protein (Adeyemi *et al.* 2021). Beyond priming, 3B recruits RdRp (3D) to initiate transcription (Ferrer-Orta *et al.* 2023). To prime positive-strand synthesis, VPg is uridylylated to form VPgpU (pU), a reaction catalyzed by 3D^pol^ in the presence of the cre RNA element (Nayak *et al.* 2005). Among the three 3B copies, 3B1 plays the most critical role in recruiting 3D by binding symmetrically at the base of the palm subdomain, connecting two 3D^pol^ molecules (Ferrer-Orta *et al.* 2023).

SARS-CoV-2 non-structural protein 1 (NSP1) inhibits host translation and promotes host mRNA degradation (Finkel *et al.* 2021). For SARS-CoV NSP1, ribosome binding and specific residues (R124, K125) are essential for its mRNA degradation activity (Fisher *et al.* 2022; Kamitani *et al.* 2009; Lokugamage *et al.* 2012). Porcine epidemic diarrhea virus (PEDV) hijacks the host RNA-binding protein Musashi homolog 1 (MSI1) via a binding site in its 3’ UTR to facilitate viral replication by shielding viral RNA from degradation (Zhou *et al.* 2025).

### Inducing membrane rearrangements to form replication compartments

SARS-CoV-2, an RNA virus, replicates in the cytoplasm. It induces the formation of double-membrane vesicles (DMVs) through virus-driven invagination and remodeling of cellular membranes (Fig. 6C) (Klein *et al.* 2020). The viral genomic RNA (+gRNA) is released from the nucleocapsid (N) protein and serves as mRNA for translating ORF1a/ORF1b, yielding NSPs. These NSPs, including the RdRp (NSP12), form replication-transcription complexes (RTCs) associated with the +gRNA.

Within DMVs, the RTC binds the 3’ end of the +gRNA and transcribes full-length negative-sense RNA (-gRNA), which then serves as a template for synthesizing new +gRNA. Post-synthesis, SARS-CoV-2 employs NSP10 to activate the proofreading (NSP14) and cap methyltransferase (NSP16) activities, alongside NSP13, for RNA capping (Krafcikova *et al.* 2020; Ma *et al.* 2015).

The newly synthesized SARS-CoV-2 +gRNA possesses a 3’ poly-A tail (median ~47 nt) (Kim *et al.* 2020). Coronavirus genomes lack a conventional polyadenylation signal; instead, the poly-A tail is added cytoplasmically by a viral poly (A) polymerase activity associated with NSP8 (Tvarogová *et al.* 2019). Poly-A tail length correlates with infection stage (~60 nt early, ~30 nt late) and influences translation efficiency (Shien *et al.* 2014; Wu *et al.* 2013). After progeny +gRNA and subgenomic RNAs (sgRNAs) are produced, they are released from DMVs. The gRNA is packaged by N protein, enveloped, and released from the cell (Brant *et al.* 2021; Hartenian *et al.* 2020).

## DISCUSSION AND CONCLUSION

Viruses employ diverse mechanisms to suppress host transcriptional activity, promoting their replication and immune evasion. They can interfere with Pol II function, inhibit host mRNA export (*e*.*g*., influenza NS1), and influence host RNA splicing and modification. For example, FMDV recruits host prolyl endopeptidase (PREP) to downregulate transcription of RIG-I and MDA5 mRNAs, directly evading cytoplasmic RNA sensing through a transcriptional mechanism (Ma *et al.* 2025). Through these strategies, viruses effectively regulate host transcription, translation, and immune responses.

Viral RNA is regulated not only by transcription factors but also by RNA modifications (*e*.*g*., m6A, pseudouridination), which control its stability, translation, and immunogenicity. While RNA modifications are recognized as important post-transcriptional regulatory mechanisms in viruses, a comprehensive understanding of how viruses specifically exploit them to regulate multiple steps is still emerging and requires systematic investigation.

Simultaneously, viruses must balance their transcriptional activity to facilitate replication while evading immune surveillance. Research into how viruses evade host immune recognition through transcriptional regulation (*e*.*g*., inhibiting interferon response, altering MHC expression) is ongoing. For example, members of the DEAD-box RNA helicase family act as host restriction factors and participate in the degradation of HIV-1 vRNA (Tacheny *et al.* 2012). However, studies that integrate immune evasion mechanisms directly with transcriptional regulatory strategies are relatively rare. Understanding the bidirectional interactions between viral transcriptional regulation and immune evasion represents an emerging frontier. Emerging technologies, such as *in situ* cryo-electron tomography and single-cell RNA sequencing, are poised to reveal the spatiotemporal dynamics of viral transcription within infected cells. For instance, single-molecule imaging has visualized the polarized translation of retroviral RNA in the cell periphery, underscoring the importance of subcellular localization in viral gene expression (Leon-Diaz *et al.* 2025). Focusing on the transcriptional mechanisms underpinning immune evasion and investigating the transcriptional “arms race” between viruses and hosts may reveal novel antiviral targets in the future.
